# Reactive oxygen species-responsive supramolecular deucravacitinib self-assembly polymer micelles alleviate psoriatic skin inflammation by reducing mitochondrial oxidative stress

**DOI:** 10.3389/fimmu.2024.1407782

**Published:** 2024-05-10

**Authors:** Leiqing Yao, Faming Tian, Qinqin Meng, Lu Guo, Zhimiao Ma, Ting Hu, Qiongwen Liang, Zhengxiao Li

**Affiliations:** ^1^ Department of Dermatology, The Second Affiliated Hospital of Xi’an Jiaotong University, Xi’an, China; ^2^ Medical Research Center, North China University of Science and Technology, Tangshan, Hebei, China

**Keywords:** psoriasis, transdermal delivery, TYK2 inhibitor, supramolecular polymers micelle, reactive oxygen species

## Abstract

**Introduction:**

The new topical formula is urgent needed to meet clinical needs for majority mild patients with psoriasis. Deucravacitinib exerts outstanding anti-psoriatic capacity as an oral TYK2 inhibitor; however, single therapy is insufficient to target the complicated psoriatic skin, including excessive reactive oxygen species (ROS) and persistent inflammation. To address this need, engineered smart nano-therapeutics hold potential for the topical delivery of deucravacitinib.

**Methods:**

hydrophobic Deucravacitinib was loaded into polyethylene glycol block-polypropylene sulphide (PEG-b-PPS) for transdermal delivery in the treatment of psoriasis. The oxidative stress model of HaCaT psoriasis was established by TNF-α and IL-17A *in vitro*. JC-1 assay, DCFH-DA staining and mtDNA copy number were utilized to assess mitochondrial function. 0.75% Carbopol^®^934 was incorporated into SPMs to produce hydrogels and Rhb was labeled to monitor penetration by Immunofluorescence. *In vivo*, we established IMQ-induced psoriatic model to evaluate therapeutic effect of Car@Deu@PEPS.

**Results:**

Deu@PEPS exerted anti-psoriatic effects by restoring mitochondrial DNA copy number and mitochondrial membrane potential in HaCaT. *In vivo*, Car@Deu@PEPS supramolecular micelle hydrogels had longer retention time in the dermis in the IMQ-induced ROS microenvironment. Topical application of Car@Deu@PEPS significantly restored the normal epidermal architecture of psoriatic skin with abrogation of splenomegaly in the IMQ-induced psoriatic dermatitis model. Car@Deu@PEPS inhibited STAT3 signaling cascade with a corresponding decrease in the levels of the differentiation and proliferative markers Keratin 17 and Cyclin D1, respectively. Meanwhile, Car@Deu@PEPS alleviated IMQ-induced ROS generation and subsequent NLRP3 inflammasome-mediated pyroptosis.

**Conclusion:**

Deu@PEPS exerts prominent anti-inflammatory and anti-oxidative effects, which may offers a more patient-acceptable therapy with fewer adverse effects compared with oral deucravacitinib.

## Introduction

Psoriasis is an immune-related chronic inflammatory disease in connection with hyperproliferation of keratinocytes and inflammatory infiltration, which is induced by immune mediators especially for IL-23/Th17 axis (1). Conventional oral therapies are often associated with various adverse events (AEs), such as hepatorenal toxicity and malignancy (2). The high economic burden, parenteral administration, risk of immunogenicity limit widespread use of biologics (3). About 70–80% of patients have localized disease, which can be controlled using topical treatments alone (4, 5). Topical agents forms the cornerstone of psoriatic treatment, including immunosuppressant, topical corticosteroids and topical vitamin D analogs (calcitriol), which may cause a toxicity with long-term usage and skin atrophy (6). Therefore, New formulas for topical agents of anti-psoriasis therapeutics are needed, which will result in more direct action on skin lesions and greater cost-effectiveness.

The overloaded reactive oxygen species (ROS) in psoriatic lesions disequilibrates the redox system (7), induces DNA oxidative damage and activates the pro-inflammatory signaling cascades (8), leading to hyperproliferation of keratinocytes (9). The excess ROS production demolish the antioxidant defence ability of cells by altering the mitochondrial genome, which further cause loss of mitochondrial membrane potential (MMP) and mtDNA damage, leading to Th17 cell response changes and IL-17 secretion (10). Targeting “activated” overloaded ROS of keratinocytes has been proposed as an effective strategy to restrain the pathogenic processes (11). Therefore, the overloaded ROS in psoriatic inflammation tissues can be exploited as a trigger for engineered nanocarriers to release their therapeutic cargo.

A new generation of small-molecule drugs have mostly been developed as oral formulas (12, 13). Deucravacitinib is an tyrosine protein kinase 2 (TYK2) inhibitor to downregulate the IL-23/Th17 and type I interferon pathway, which has been approved by the Food and Drug Administration for oral therapy of psoriasis (14–16). Importantly, deucravacitinib did not participate in cell homeostasis and hematopoiesis, lipid metabolism and granulocyte production, which showed a reduced likelihood of adverse effects compared to JAK1–3 inhibitors (17, 18). For conventional oral administration, transdermal drug delivery provides many advantages in particular with reduced first pass metabolism, enhanced therapeutic efficiency by acting directly on the stratum corneum (SC) (19). However, no topical formulation of deucravacitinib has been developed to date, which is expected to reduce the dosage and adverse effects associated with its oral administration such as upper respiratory tract infection and nasopharyngitis (20).

However, because of the lipophilic property of Deucravacitinib, it is difficult to penetrate the complex hydrophobic and hyperproliferative SC barrier, which is the primary challenge in the development of a topical therapeutic agent for psoriasis (21, 22). These challenges can be addressed with the appropriate drug nanocarriers with effective targeting and extended residence time at the lesion site. For example, biomimetic iron single-atom catalysts (FeN_4_O_2_-SACs) (23) is used for psoriasis treatment for ROS-responsive capacity. Supramolecular polymers micelles (SPMs) are occured by the self-assembly of monomer units (24–26) and formed by intermolecular interactions. Such polymerization usually relies on non-covalent interactions such as pi-pi stacking, hydrophobic interactions, etc. The process of SPMs are achieved by the adjustable solution pH (27), stimulus responsiveness or ionic strength (28–30).

**Figure 1:**
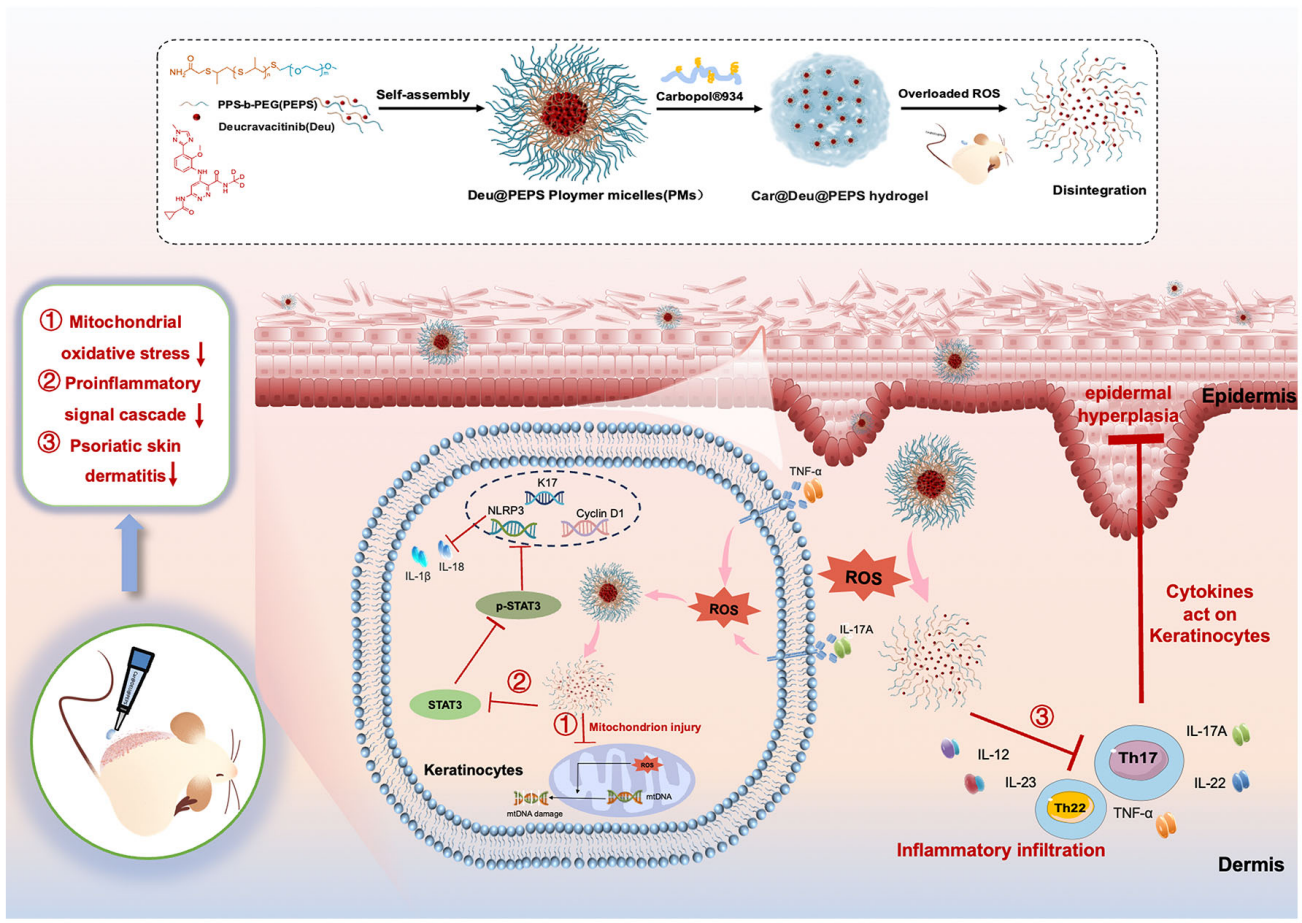
Schematic diagram of the Car@Deu@PEPS hydrogel with ROS scavenging capacity in the treatment of psoriasis.

In this study, to control the biodistribution of Deucravacitinib targeting excessive ROS in the psoriatic SC layer, we synthesized Deucravacitinib-loaded poly(ethylene glycol)-b-poly(propylene sulfide) (PEG-b-PPS) diblock copolymers (Deu@PEPS). The amphiphilic PEG-b-PPS (PEPS) enfold lipophilic Deucravacitinib within the hydrophobic bilayer and allow therapeutic drugs to be released on demand at specific sites of action via oxidation conversion of nanocarrier (31, 32). Hence, we demonstrate an application of Deu@PEPS hydrogel for coinstantaneous anti-inflammatory and antioxidant synergistic therapies in psoriasis (**Figure 1**). We confirmed the characteristics and ROS-responsive reaction of Deu@PEPS to validate the principal for targeting the oxidative damage site of the psoriatic skin. We further evaluated the permeability and the therapeutic effects *in vivo* using an imiquimod (IMQ)-induced psoriasis mouse model and using oxidative stress-stimulated HaCaT cells *in vitro*. This study can provide a new therapeutic strategy to improve the efficacy of deucravacitinib, and potentially other JAK inhibitors, offering a highly efficient anti-inflammatory and antioxidant synergistic therapy in the future.

## Materials and methods

### Synthesis and characterization of deucravacitinib-loaded PEPS supramolecular polymers micelles

PEPS was synthesized with few amendment according to a previously published literature (33, 34). ^1^H nuclear magnetic resonance (1H NMR) of PEPS were recorded by spectroscopy with CDCl3 (Agilent, USA). Deucravacitinib was purchased from Fu Ruidi Biotech Co., Ltd (Wuhan, China). 2 mg of PEPS and 20 mg of deucravacitinib (dissolved in DMSO) was slowly added to double distilled water. Next, the solvent was removed by rotary evaporation, washing and dialysis (MWCO = 3 kDa) every 6–8 h for two days. Rhodamine and PEG-b-PPS were dissolved in 10 ml dichloromethane as the oil phase and 20 ml of sodium cholate as the water phase. The oil phase was dropped into the water phase followed by 1min ultrasound, and the dichloromethane was completely removed using rotary evaporation. Then 10 ml sodium cholate was added and diluted, centrifuged at 12000 rpm for 30 min, and finally resuspended in deionized water. Ultraviolet and fluorescence spectroscopy showed effectiveness of Rhodamine markers. Deu@PEPS-Rhb was prepared similarly to Deu@PEPS except for performing in the darkness environment.

The morphology of the SPMs were observed by transmission electron microscopy (TEM), performed on a Talos F200X. Zeta potential and size distribution were detected by dynamic light scattering (DLS, Malvern Zetasizer system). Entrapment efficiency (EE%) and Drug Loading capacity% (DL%) were also examined by highperformance liquid chromatography (HPLC). EE% = Weight of deucravacitinib in polymer micelles/Weight of the feeding × 100%; DL% = Weight of deucravacitinib in polymer micelles/Weight of the feeding deucravacitinib plus polymer × 100%. All measurements were in triplicate. Nicole 50 Fourier transform infrared spectroscopy (FTIR) recorded Deu@PEPS and oxidative product of PEPS from 3500 to 500 cm^−1^.

### *In vitro* release assay

Different concentrations of Deu@PEPS containing H_2_O_2_ (5 mM or10 mM) or PBS were utilized to assess the ROS-responsive capacity by HPLC. For oxidation sensitive drug release, deucravacitinib release profiles were conducted in 20 ml PBS. Dialysis tube was used to collect free Deucravacitinib. 1 ml of the solutions was evacuated at 0, 1, 2, 4, 8, 12, 24 h, which were detected by HPLC at the λ max (254 nm).

### Cell culture and treatment

Immortalized human keratinocytes (HaCaT cells), purchased from the China Center for Type Culture Collection, were cultivated in RMPI 1640 medium and complemented with 10% Fetal bovine serum (Gibco, USA), penicillin and streptomycin (NCM, China). HaCaT cells were incubated in 5%CO_2_ circumstance at 37°C. HaCaT cells were co-stimulated with 25 ng/ml TNF-α and 50 ng/ml IL-17A (Abclonal, China) for 24 h in order to induce psoriatic inflammation and oxidative stress.

### *In vivo* biodistribution and permeability of Car@Deu@PEPS in mice

Wild mice C57BL/6 (female, 6-8 week old) were obtained from GemPharmatech Co.,Ltd. Mice were hosted in a controlled environment with specific-pathogen-free condition. We used C57BL/6 mice to establish the psoriasiform skin inflammation model because this strain is proven to provide a better genetic background for the pathogenesis of psoriasis than other strains (35). C57BL/6 mice were divided into control and IMQ groups. After 6-days 62.5 mg imiquimod (IMQ) cream (Mingxin Sichuan, China) was applied to the shaven dorsal skin to induce psoriatic dermatitis. Carbopol^®^934 was prepared by neutralizing the gel to pH 6 by triethanolamine. 0.75% Carbopol^®^934 was chosen as the matrix owing to ease in utilization and enough viscosity for topical application. Car@Deu@PEPS-Rhb were topically used in back skin (1 mg/kg) once and mice were sacrificed at different time intervals. The permeation of Car@Deu@PEPS-Rhb in psoriatic lesions was detected by immunofluorescence. The distribution of Car@Deu@PEPS-Rhb was imaged using *In vivo* imaging system (IVIS) at 24h, 48h, 72h time points to evaluate accumulation in different organs. *Ex vivo* optical imaging were conducted using black paper. All of the above performed in a dark environment.

### *In vitro* cellular uptake

Cellular uptake was performed by HaCaT keratinocytes. Rhb labeled PEPS was used to loading deucravacitinib for fluorescence imaging. Cells were paved onto 12-well flat-bottomed plates (3 × 10^5^ cells/well). After cell adherence, 0.5 μg/ml Deu@PEPS or 0.5μg/ml deucravacitinib were added and pre-incubated for 12h. Next, cells were dealed with 200 μM H_2_O_2_ (incubated for 6 h) or TNF-α and IL-17A (incubated for 24 h) to stimulate ROS. The whole experiment was conducted keep out of the light.

### CCK8 assay

HaCaT keratinocytes (5 × 10^3^ cells/well) were plated until cell attachment. For toxicity detection, PEPS or Deu@PEPS (0-400 μg/ml) were cultured for 48 h. Next, HaCaT cells were treated with 10% CCK8 (NCM, China) and incubated. Cell viability was accessed by the microplate reader. For cell proliferation assay, the time when cells adhered to the wall was recorded as point 0, then IL-17A + TNFα and different concentration of Deu@PEPS or deucravacitinib were incubated continued (24, 48, and 72 h). Finally, the cells were managed with CCK8 and calculated as we performed before.

### Detection of intracellular ROS and mitochondrial membrane potential *in vitro*

Fluorescent probes 2′,7′-Dichlorodihydrofluoresce in diacetate (Solarbio, China) were used to detect intracellular ROS in cells. DCFH-DA as oxidative sensitive fluorescent probe allowed to assess specific intracellular ROS by being oxidised to fluorescent DCF (36).The loss of MMP was estimated by JC-1 kit according to manufacturer’s introduction (Beyotime, China). To perform assays, HaCaT cells (2 × 10^5^ cells/well) were paved overnight. Then, the cells were pretreated with deucravacitinib or Deu@PEPS (0.5 μg/ml)and co-stimulated by TNF-α and IL-17A for 24 h. The cells were dyed with JC-1 dye or DCFH-DA probes (10 μM) in the sunblock and washed to remove remaining probes. The staining was tested using the fluorescence microscope.

### Mitochondrial DNA assay

Real-Time PCR was utilized to estimate the mtDNA copy number (37). DNA was isolated from psoriatic HacaT cells with DNA extraction kit (Beyotime, China). Normalizing mtND1 gene levels to nuclear beta-2 microglobulin levels were used to access mtDNA copy number (38). Genomic DNA was amplified by elongase polymerase primers (KOD FX, Toyobo, Japan) and long mtDNA fragments were detected (**Additional file 2: Table S2**)

### Imiquimod-induced psoriasis like mouse model

After 7 days of adaptation in the SPF environment, the hair of 6-8 week female mice C57BL/6 back skin was shaved (n = 6-7 per group). Mice were normally fed for 1 day to restore the stratum corneum, 62.5 mg of IMQ was applied to induce psoriatic dermatitis for 6 consecutive days. Psoriasis Area and Severity Index (PASI) scores for thickening, erythema and scaling were determined as reported (39). Body weights changes of mice were recorded. After mice euthanization, spleen samples were weighed, photographed and spleen/body wt% was measured. Skin tissues were quick freezed by liquid nitrogen. Finally, to evaluate short-term biocompatibility of hydrogels *in vivo*, mice blood samples were used to assess hepatorenal function by biochemical detection.

### Hematoxylin and eosin staining

Tissues of mice skin and organs were fixed with formaldehyde. Then tissues were sectioned at 7-10 μm for H&E staining, immunohistochemistry and immunofluorescence. H&E staining was conducted for evaluation of epidermal hyperplasia, skin inflammation and major organ toxicity. Means of epidermal thickness were counted using Image J.

### Immunohistochemistry and immunofuorescence analysis

Tissue sections were stained with Ki67 (GB121141, Servicebio, China), CD3 (GB11014, Servicebio, China), myeloperoxidase (GB15224, Servicebio, China) for IFC and STAT3 (10253, proteintech, China), Krt17 (A3769, Abclonal, China), Cyclin D1 (A0310, Abclonal, China) for IF. Slices were scanned using 3DHISTECH.

### Dihydroethidium staining *in vivo*

DHE can be oxidised into red fluorescence because it is sensitive to reactive oxygen species (40). To evaluate the ROS levels in IMQ induced skin lesion, skin samples were collected after mice were sacrificed and were frozen instantaneously. The frozen sections were stained with DAPI (Beyotime, China) and DHE (Servicebio, China) at 37°C for 30 mins.

### The anti-oxidative assay

The H_2_O_2_ scavenging power of PEPS to ROS was evaluated by ABTS radical cation scavenging activity test (T-AOC). The scavenging ability of Deu@PEPS in HaCaT was evaluated after co-stimulated with TNF-α + IL-17A for 24 h by T-AOC and SOD. The levels of T-AOC, SOD, MDA and GSH-Px in mice skin and plasma were detected by assay kits according to the manufacture’s guideline (Nanjing JianCheng, China).

### Terminal deoxynucleotidyl transferase dUTP nick-end labelling staining

TUNEL assay was utilized to monitor *in situ* DNA fragmentation. Mice skin lesions were embedded in optimal cutting temperature compound and stained with the TUNEL kit (Servicebio, China).

### RNA extraction and quantitative real-time polymerase chain reaction

20 mg RNA of mice skin samples (physical homogenization) and cells were extracted by TRIzol (Invitrogen, USA). After chloroform extraction, isopropyl alcohol was added and allowed to stand before centrifugation. Next, the RNA was washed with ethanol, centrifuged and dried, and 25 μL DEPC water was added. The purity and concentration of RNA were measured by NanoDrop (ThermoFisher, USA). Genomic DNA removal was performed and complementary DNA was reversed transcribed (Accurate Biology, China). Quantitative realtime polymerase chain reaction (PCR) was performed by SYBR Green method (TransGen Biotech, China) using RT-PCR System (Bio-Rad, CA). Primers are described in the (**Additional file 2**: **Table S3**). The mRNA expression levels were figured by 2^−ΔΔCT^ method. Data were normalized to β-actin or GAPDH and compared with controls.

### Evaluation of skin irritation and long term cytotoxicity

Different formulas were used on the back of C57BL/6 mice continuing 28 days and control group only managed with Carbopol^®^934 (n = 4 per group). The weight of the mice was documented and photographed before the hydrogel was given. Signs of skin irritation were monitored as reported before (41). Scratching bouts were assessed every 7 days. After 28 days, mice were euthanized after orbital vein blood collection under anesthesia. Spleen samples were weighed, photographed and spleen/body wt% was measured as the index of immune activation. Long term biocompatibility *in vivo* was evaluated like short term before.

### Statistical analysis

Statistical analyses were conducted by GraphPrism 9.0. The data are shown as the mean ± standard deviation (Mean ± SD). One-way or two-way analysis of variance was utilized for multiple comparisons. If not specifically requested, p < 0.05 was considered statistically significant.

## Results

### Physicochemical characteristics and oxidation-responsive drug release of Deu@PEPS SPMs

**Figure 2:**
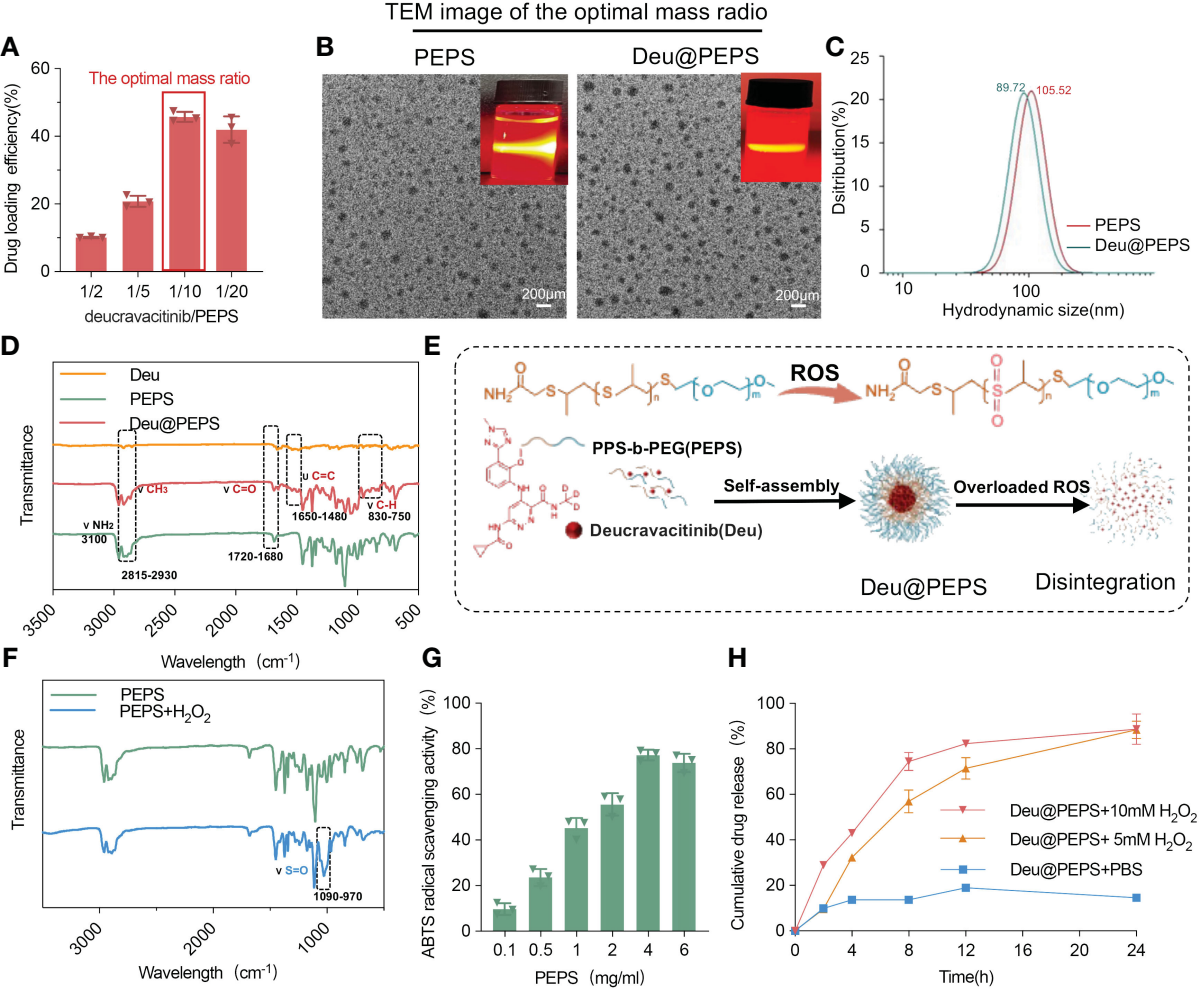
Characteristization and ROS-responsive drug release of Deu@PEPS. **(A)** The loading efficiency of drug within different mass radio of deucravacitinib/PEPS as determined by HPLC. **(B)** TEM images and Tyndall phenomenon (upper right corner) of PEPS and Deu@PEPS (scale bar: 200 nm). **(C)** The average hydrodynamic diameter of PEPS and Deu@PEPS detected by DLS. **(D)** FTIR spectroscopy of Deu@PEPS. **(E)** Schematic illustration of the preparation of PEG-b-PPS loaded deucravacitinib (Deu@PEPS) and the ROS-responsive action. **(F)** FTIR spectroscopy of PEPS and its oxidative products under H_2_O_2_ stimulation. **(G)** ROS-scavenging activities of PEPS at different concentrations (0.1, 0.5, 1, 2, 46 and 6 μg/ml) by ABTS radical cation scavenging activity assay. **(H)** *In vitro* drug release of Deu@PEPS under different concentration of H_2_O_2_ or PBS detected by HPLC (n = 3). FTIR, Fourier-transform infrared spectroscopy; HPLC, highperformance liquid chromatography; DLS, dynamic light scattering; TEM, ransmission electron microscopy.

The diblock polymerization of PEPS was characterized by the peaks in the ^1^H nuclear magnetic resonance (NMR) spectrum (**Additional file 1: Figure S1**): ^1^H NMR (chloroform-d) δ 3.64 (s, 4 mH, OCH_2_CH_2_), 3.49–3.44 (m, 3H, CH_3_), 2.91–2.62 (d, nH, SCHCH_2_), 1.36 (d, 3nH, CH_3_). The mass ratio of 10/1(PEPS to deucravacitinib) was ultimately selected on the basis of the higher drug encapsulation efficiency (~45.76%) and drug loading rate (~4.16%) (**Figure 2A**), as determined by HPLC. PEPS and Deu@PEPS both showed the Tyndall phenomenon and a spherical in morphology by TEM (**Figure 2B**). DLS exhibited a uniform size distribution, which showed the average hydrodynamic size of the PEPS and Deu@PEPS was 89 nm and 105 nm (**Figure 2C**). Fourier-transform infrared spectroscopy (FTIR) showed a slight peak at 750–830 cm^–1^, suggesting the ionization of the benzene ring structure. The addition of deucravacitinib led to conformation changes of the enhanced carbon-carbon and carbon-oxygen double bond vibration at 1480–1650 cm^-1^ and 1680–1720 cm^-1^, respectively. CH_3_ stretching vibration was also enhanced at 2815–2930 cm^–1^, indicating that deucravacitinib was successfully loaded on the PEPS (**Figure 2D**). We also synthesized rhodamine b-labeled PEPS (Rhb-PEPS) as a fluorescence probe for monitoring (**Additional file 1: Figure S2**, **Additional file 2: Table S1**).

Next, H_2_O_2_ was selected to assess the ROS-responsive reaction and drug release behavior (**Figure 2E**). The appearance of an absorption band at 970–1090 cm^−1^ in the PEPS products indicated conversion of the S=O group to poly (propylene sulphone) after H_2_O_2_ stimulation (**Figure 2F**). We further evaluated the antioxidant activity of PEPS by a 2,2-azinobis (3-ethylbenzothiazoline-6-sulfonic acid) (ABTS) activity. The PEPS manifested significant clearance of ABTS radical cations in the concentration range of 0.1 to 4.0 mg/ml in a dose-dependent pattern (**Figure 2G**). Finally, The drug release was faster at 0-4 h (43.08% ± 2.08%) under 10 mM H_2_O_2_ than under 5 mM H_2_O_2_ (32.31% ± 1.31%). At 24 h, 91.1% ± 7.70% of the deucravacitinib had been released, which was significantly higher than the amount released in the absence of H_2_O_2_ (14.67% ± 0.3%) (**Figure 2H**). DLS further confirmed the stability of storing the SPMs at room temperature for 1 month by assessing the hydrodynamic diameter (**Additional file 1: Figure S3**).

### Cell uptake, penetration, and biodistribution of Deu@PEPS

**Figure 3:**
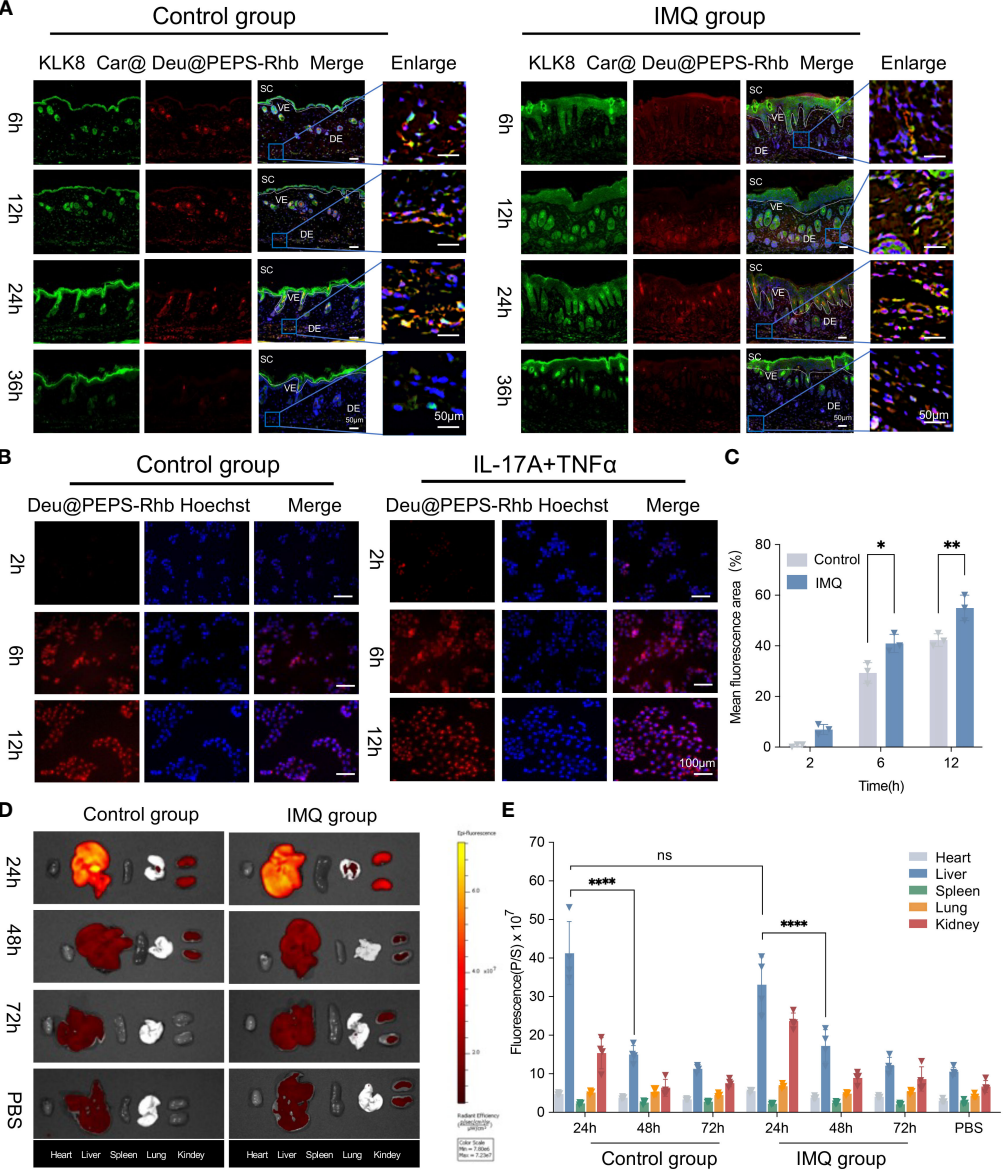
Cell uptake, biodistribution and penetration of Deu@PEPS. **(A)** The penetration of Car@Deu@PEPS-Rhb (red) through control and IMQ-induced psoriatic skin at 6, 12, 24 and 36 h by Immunofluorescence staining (IF). (scale bar: 50 μm). KLK8 (green) was stained to differentiate the SC layer. **(B)** Fluorescence microscopic images of cell uptake using Car@Deu@PEPS-Rhb at interval time points between control and IL-17A+TNFα co-stimulated group in HaCaT cells. **(C)** Quantitative analysis of relative fluorescence area% (n = 3). **(D)** *In vivo* imaging system (IVIS) image of mice organs after Car@Deu@PEPS-Rhb (1.0 mg/kg) at different time points (24, 48 and 72h) between control and IMQ groups. **(E)** Quantitative analysis of average radiant efficiency in different organs (n = 4). Car, Carbopol^®^934; SC, stratum corneum; VE, visible epidermal; DE, dermis. *∗∗∗∗p < 0.0001, ns means no significant differences*.

To facilitate *in vivo* skin permeability performance and quantification, Deu@PEPS was labeled with Rhb fluorescent dye and administered topically to IMQ-induced mouse model, which has been widely used for psoriatic physiopathology and drug development owing to its strong dependence on the IL-23/IL-17 axis (39). The SPMs were incorporated in 0.75% Carbopol^®^934 to produce the hydrogel (Car@Deu@PEPS-Rhb). A single dose of Car@Deu@PEPS-Rhb (1 mg/kg) was topically administered to the dorsal skin. Immunofluorescence (IF) revealed the similar red fluorescence pattern between the control and IMQ groups under the SC and visible epidermal layer at 6 h. Both groups displayed the strong fluorescent signal in the dermis, which increased in a time-dependent manner during 24 h. After 36 h administration, the deposition of Deu@PEPS remained distinct in the dermis of the IMQ group but was negligible in the control group (**Figure 3A**), indicating enhanced skin retention in the oxidative stress microenvironment of the psoriatic skin. Meanwhile, TNF-α and IL-17A were utilized to establish the psoriasiform and oxidative stress HaCaT model. Red fluorescence was first observed in the TNF-α and IL-17A stimutated group as early as 2 h rather than control group. At 6 h, the fluorescent signal was mainly concentrated around the cells, whereas it was concentrated in the center of the cells at 12 h of incubation (**Figures 3B, C**), indicating earlier cell uptake of the SPMs by TNF-α and IL-17A stimulation.

Finally, The major organs were collected at different times after topical administration. The biodistribution of Car@Deu@PEPS-Rhb in the major organs showed that the SPMs mainly accumulated in liver and kidney. But we observed no significant difference between the IMQ and control groups at 24 h in liver in both groups (p = 0.6874). Meanwhile, both two groups showed a similar metabolism rate in liver from 24 to 48 h (p < 0.0001) (**Figures 3D, E**). By 72 h, the fluorescence intensities in the liver and kidneys of the two groups were similar to those of the PBS group, which manifested a nearly consistent metabolic profile in both two groups.

### Deu@PEPS exerts anti-psoriatic effects by reestablishing mitochondrial function

**Figure 4:**
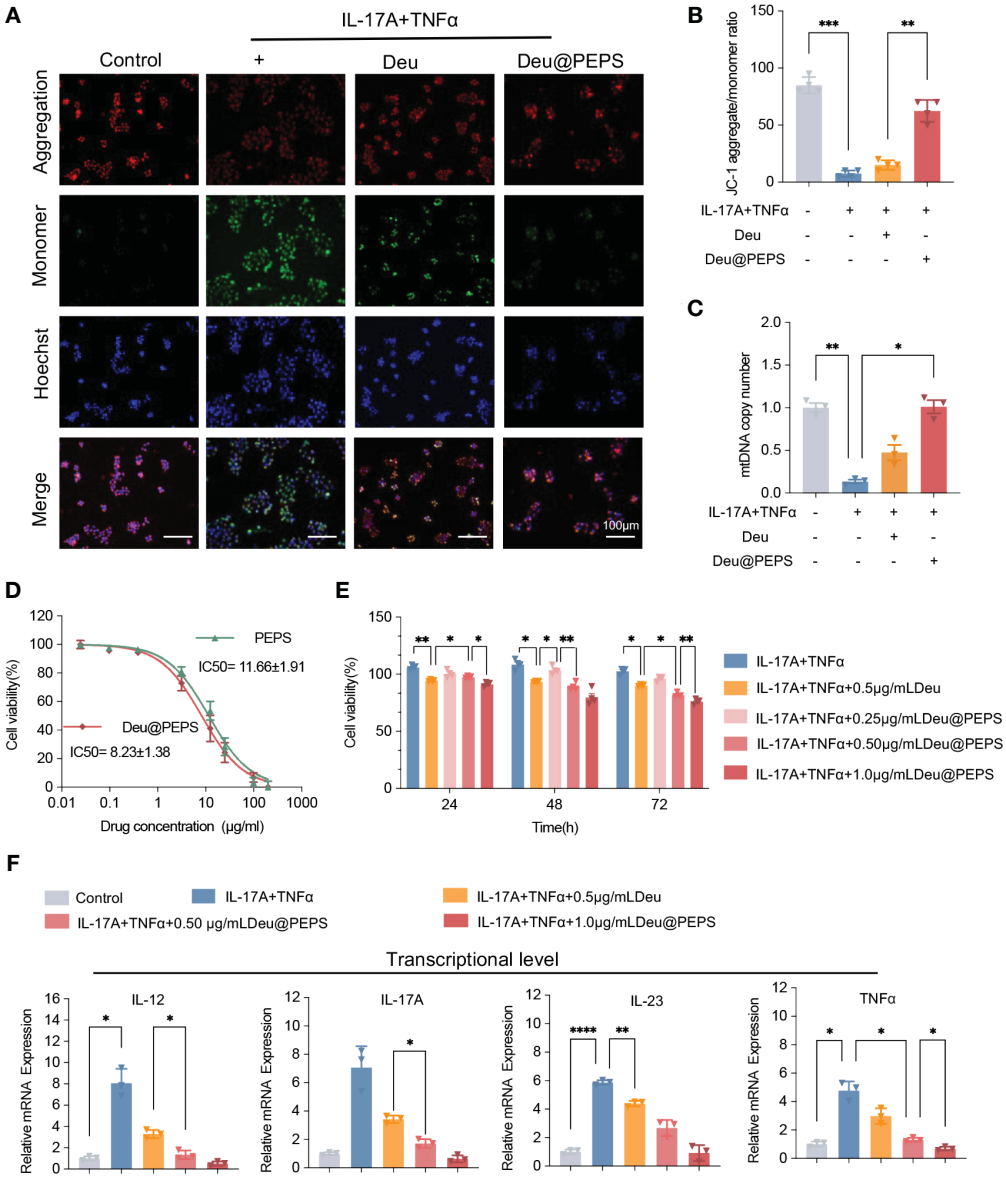
Deu@PEPS alleviates oxidative stress-induced mitochondrial dysfunction to exert anti-psoriatic effects *in vitro.* **(A)** Representative fluorescence microscopic images of JC-1 staining indicated the loss of mitochondrial membrane potential (scale bar: 100 μm). **(B)** Quantitative JC-1 staining aggregate/monomer ratio. **(C)** Levels of mtDNA copy number of HaCaT cells. **(D)** Cytotoxicity of PEPS and Deu@PEPS in HaCaT cells determined by CCK8 assay (n = 4). **(E)** Cell proliferation in HaCaT cells by pretreatment with Deu@PEPS (0.5 or 1.0 μg/ml) or deucravacitinib (0.5 μg/ml) for 12 h and stimulated with TNFα and IL-17A then incubated for 24 h. **(F)** Relative mRNA expression of *IL-12, IL-17A, IL-23* and *TNF-α* in HaCaT (n = 3). *∗p< 0.05, ∗∗p < 0.01, ∗∗∗p < 0.001, ∗∗∗∗p < 0.0001*.

To confirm the therapeutic effect of Deu@PEPS to restrain the overproduction of ROS to control the inflammation in keratinocytes, we used 2′,7′-dichlorofluorescein diacetate (DCFH-DA) to detect introcellular ROS level. Firstly, Intracellular ROS level were elevated after 200 μM H_2_O_2_, which were similar to TNF-α and IL-17A co-stimulation. Deu@PEPS exhibited a lower intensity of green fluorescence compared with control group, confirming the ability of the SPMs to obliterate excessive ROS in psoriatic keratinocytes (**Additional file 1: Figure S4**). T-AOC and SOD levels of HaCaT cells decreased after stimulation with TNF-α and IL-17A, but increased after treatment with Deu@PEPS (**Additional file 1: Figure S5**). In comparison with control group, the psoriasiform HaCaT group showed negligible red fluorescence and enhanced green fluorescence, manifesting the loss of MMP. However, Deu@PEPS (0.5 μg/ml) recovered mitochondrial polarization under oxidative stress, which manifested as strong red fluorescence and an increased ratio of red/green fluorescence (**Figures 4A, B**). The mtDNA copy number was reduced in HaCaT cells after TNF-α and IL-17A, and this depletion was reversed with Deu@PEPS pretreatment, but not with treatment of deucravacitinib alone, suggesting the ability of PEPS to prevent mtDNA damage (**Figure 4C**). Taken together, our results suggest that Deu@PEPS has antioxidant properties to rescue oxidative stress-caused mtDNA damage and mitochondrial dysfunction.

We next investigated the anti-psoriatic effect of Deu@PEPS among the keratinocytes. PEPS and Deu@PEPS exhibited gradual concentration-dependent cytotoxicity with a half-maximal inhibitory concentration value of 11.66 ± 1.91 μg/ml and 8.23 ± 1.38 μg/ml, respectively (**Figure 4D**). The co-stimulation of TNF-α and IL-17A significantly increased proliferation over time. However, pretreatment of Deu@PEPS resulted in a decrease in cell proliferation in a dose-dependent manner from 24 to 72h. 0.5 μg/ml Deu@PEPS exhibited more powerful anti-proliferative capacity at 72 h compared to the same dose of deucravacitinib alone (**Figure 4E**). Quantitative real-time polymerase chain reaction (qRT-PCR) showed that TNF-α and IL-17A stimulation increased the mRNA expression of the inflammatory cytokines (**Figure 4F**), whereas Deu@PEPS pretreatment decreased their expression levels at both doses, and 0.5 μg/ml deucravacitinib alone also showed a slight but significant attenuating effect (*p<0.05*).

### Deu@PEPS significantly reduces IMQ-induced psoriatic dermatitis and normalizes the epidermal structure *in vivo*

Before evaluating the therapeutic effects of Deu@PEPS *in vivo*, a pilot study was carried out to determine the optimal dose or treatment interval of the Deu@PEPS. Compared to the IMQ group, the free deucravacitinib (5 mg/kg, QD) and blank PEPS (10 mg/kg, QD) groups showed similar effective anti-psoriatic effects. However, these effects were inferior to those of Deu@PEPS (0.45 mg/kg deucravacitinib, BID), which exhibited the optimum therapeutic effect to improve the skin clinical phenotype (**Additional file 1: Figure S6**). H&E staining showed conspicuous depressed epidermal thickness and infiltration with inflammatory cells after treatment with Deu@PEPS (0.45 mg/kg deucravacitinib, BID). This result was consistent when considering the effect on total PASI scores. Our preliminary study showed that a low dose of Deu@PEPS (0.45 mg/kg deucravacitinib, BID) was more effective than a 11.11-times higher dose of deucravacitinib alone (5 mg/kg, QD). The increased spleen body wt% is another symbol of psoriasis severity, which reflects increased immune activation in spleen (39, 42). Deu@PEPS resulted in a reduction in IMQ-induced splenomegaly; however, there was no significant change of spleen body wt%, which is likely due to individual differences and the small number of mice.

**Figure 5:**
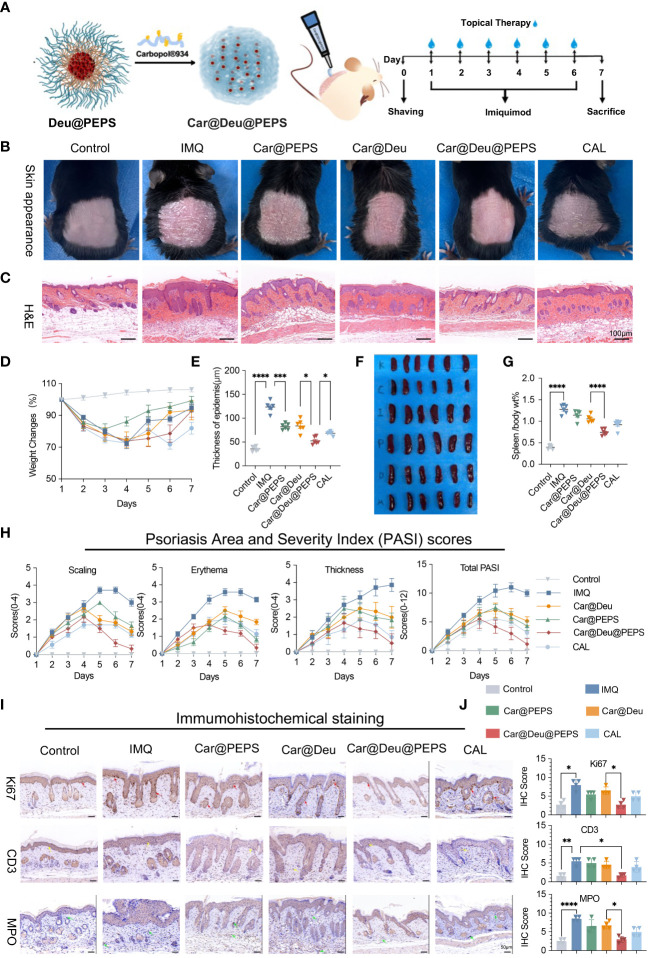
Topical application of Car@Deu@PEPS hydrogel improves IMQ-induced psoriasis like skin inflammation. **(A)** Schematic illustration of IMQ-induced psoriatic dermatis establishment and therapeutic process (n = 6-7 per group) including control, IMQ, Car@PEPS, Car@Deu (1 mg/kg deucravacitinib), Car@Deu@PEPS (0.45 mg/kg deucravacitinib), CAL (positive control) groups. **(B)** Representative skin pictures of mice to treatment with various formulas on day 7 before harvesting back skin. **(C)** Hematoxylin and eosin staining (H&E) staining of day 7-harvest mouse skin sections (scare bar: 100 μm). **(D)** Weight changes during 7 days of different groups. **(E)** Thickness of epidermis on H&E staining of different groups analysed (n = 5). **(F)** Representative photos of spleen appearance after sacrificed. **(G)** The radio of spleen weight to body (spleen body wt%) of different groups (n = 6). **(H)** Psoriasis Area and Severity Index (PASI) scores of different groups including scaling, erythema, thickness and total PASI. **(I)** Immumohistochemical (IHC) staining of Ki67 positive cells (proliferative cells, red arrows), CD3 positive cells (infiltrating T cells, yellow arrows) and MPO positive cells (neutrophils, green arrows) in skin lesions with different treatments (n = 3, scale bar: 50 μm). **(J)** IHC scores of Ki-67, CD3 and MPO with the different treatments (n = 3). IMQ, imiquimod; Car, Carbopol^®^934; Calcipotriol,CAL. *∗p< 0.05, ∗∗p < 0.01, ∗∗∗p < 0.001, ∗∗∗∗p < 0.0001*.

**Figure 5A** shows a schematic diagram of the synthesis and transdermal delivery for the treatment of psoriatic dermatitis. The IMQ-induced macroscopic scaly and thickened skin was apparently reduced of various treatment groups (BID) to some extent. In particular, Car@Deu@PEPS resulted in a similar skin appearance to that of the control group, which was superior to a 2.22-times higher dose of Car@Deu (**Figure 5B**). To our surprise, Car@PEPS group attained the greatest and earliest weight recovery, which was even better than that of the Car@Deu@PEPS group (**Figure 5D**, **Additional file 1: Figure S7**). Consistent with the improvements in skin appearance, H&E staining showed the greatest improved parakeratosis and epidermal acanthosis in Car@Deu@PEPS group (**Figure 5C**). The epidermal thickness was reduced by 33.4%, 57.4%, and 44.6% in the Car@Deu, Car@Deu@PEPS and CAL groups, respectively (**Figure 5E**). Car@Deu@PEPS group showed the most significantly atrophied spleens and diminished spleen body wt% after IMQ treatment, similar to those of the CAL group, as we expected (**Figures 5F, G**). After 7 days’ experiment, Car@Deu@PEPS group obtained the lowest total PASI (1.16 ± 0.98) and IMQ group gained the highest total PASI (10.50 ± 0.56). By contrast, the scaling, erythema, and thickness scores were increased in the Car@PEPS and Car@Deu@PEPS group initially, which was likely due to the fact that the less ROS was insufficient to induce micelle disintegration at the beginning (**Figure 5H**). Tissue samples of organs and blood were collected to assess the short-term toxicity. As shown in **Additional file 1: Figure S8**, no apparent changes of histological and serum biochemical indicators were observed, indicating that these hydrogels only cause negligible toxicity.

To confirm whether continuous topical application could prevent psoriatic pathologic hallmarks, immunohistochemistry was performed to evaluate hyperproliferating keratinocytes and inflammatory infiltration (**Figures 5I, J**). Staining of the hyperproliferating marker Ki67 was enhanced along the basal layers of the epidermis in IMQ group. However, the number of Ki67-positive cells and the staining intensity were reduced to the greatest extent in the Car@Deu@PEPS group compared to those of the IMQ group (red arrows). A cluster of neutrophils (MPO^+^, green arrows) was found in the dermis of the IMQ group and neutrophil infiltration was decreased to the greatest extent in the Car@Deu@PEPS group. Moreover, the Car@Deu@PEPS hydrogel alleviated the infiltration of T cells (CD3^+^, yellow arrows), particularly in the dermis layer.

### Deu@PEPS regulates ROS accumulation and exerts oxidative resistance *in vivo*

**Figure 6:**
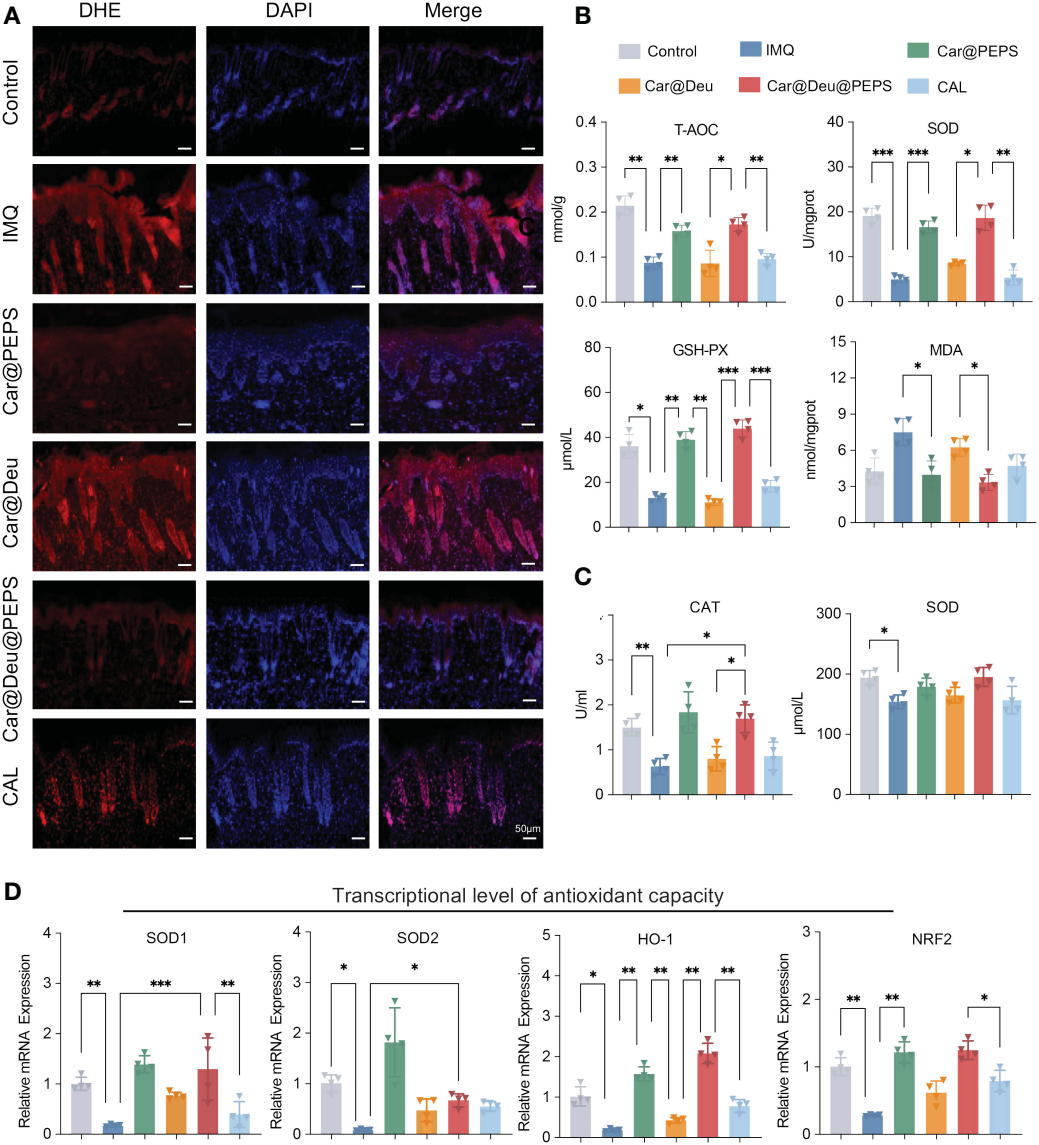
Deu@PEPS regulates ROS accumulation and exerts oxidize resistance *in vivo.* **(A)** Representative images of the fluorescence assay of ROS in the IMQ-induced skin lesions using a DHE (red) probe on day 7 (n = 3, scale bar: 50 μm). **(B)** Skin antioxidant levels of antioxidant capacity (T-AOC), superoxide dismutase (SOD) and glutathione peroxidase (GSH-Px) and lipid peroxidation product Malondialdehyde (MPA) levels of mice skin. **(C)** The plasma antioxidant levels of Catalas (CAT) and SOD. **(D)** Relative mRNA expression level of *SOD1*, *SOD2*, *HO-1* and *NRF2* of mice skin in six groups (n = 4). *∗p < 0.05, ∗∗p < 0.01, ∗∗∗p < 0.001*.

Whether Deu@PEPS reduces ROS overload in psoriatic lesions *in vivo* needs to be further explored by Dihydroethidium (DHE) staining. Compared to the excessive ROS accumulation detected in the IMQ group, DHE fluorescence intensely was substantially reduced in the Car@Deu@PEPS and Car@PEPS groups, with only slight reduction detected in the Car@Deu and CAL groups (**Figure 6A**). These results indicated that PEPS could decrease the generation of ROS in psoriatic skin, which is attributed to the conversion of the S=O group of PEPS nanocarriers. To evaluate whether topical administration influenced antioxidant enzyme system, we monitored the levels of oxidative stress markers and the lipid peroxidation product Malondialdehyde (MDA), for intracellular metabolism fatty acid oxidation could be exacerbated of psoriasis (43). The levels of T-AOC, SOD, and GSH-Px in skin lesions were significantly reduced after the induction of psoriatic dermatitis. And these reductions were markedly blocked by treatment of Car@Deu@PEPS and Car@PEPS. In contrast, MPA was increased in the lesions of the IMQ model group and decreased following Car@Deu@PEPS treatment (**Figure 6B**). The plasma antioxidant oxidase levels (catalase, SOD) also decreased after Car@PEPS and Car@Deu@PEPS treatment, although the decrease in SOD levels was not statistically significant (**Figure 6C**).

Finally, qRT-PCR demonstrated that IMQ topical application profoundly decreased the relative mRNA expression of *SOD1*, *SOD2*, *HO-1* and *NRF2*, which were improved to the greatest extent by Car@PEPS treatment, followed by Car@Deu@PEPS treatment, showing similar levels to those of the control group. CAL and Car@Deu showed slight antioxidative capability, but there was no significant difference in the levels of the antioxidant-related genes from those of the IMQ group (**Figure 6D**).

### Deu@PEPS antagonizes K17/Cyclin D1 expression through suppressing ROS−induced STAT3 and improving the transcriptional phenotype

**Figure 7:**
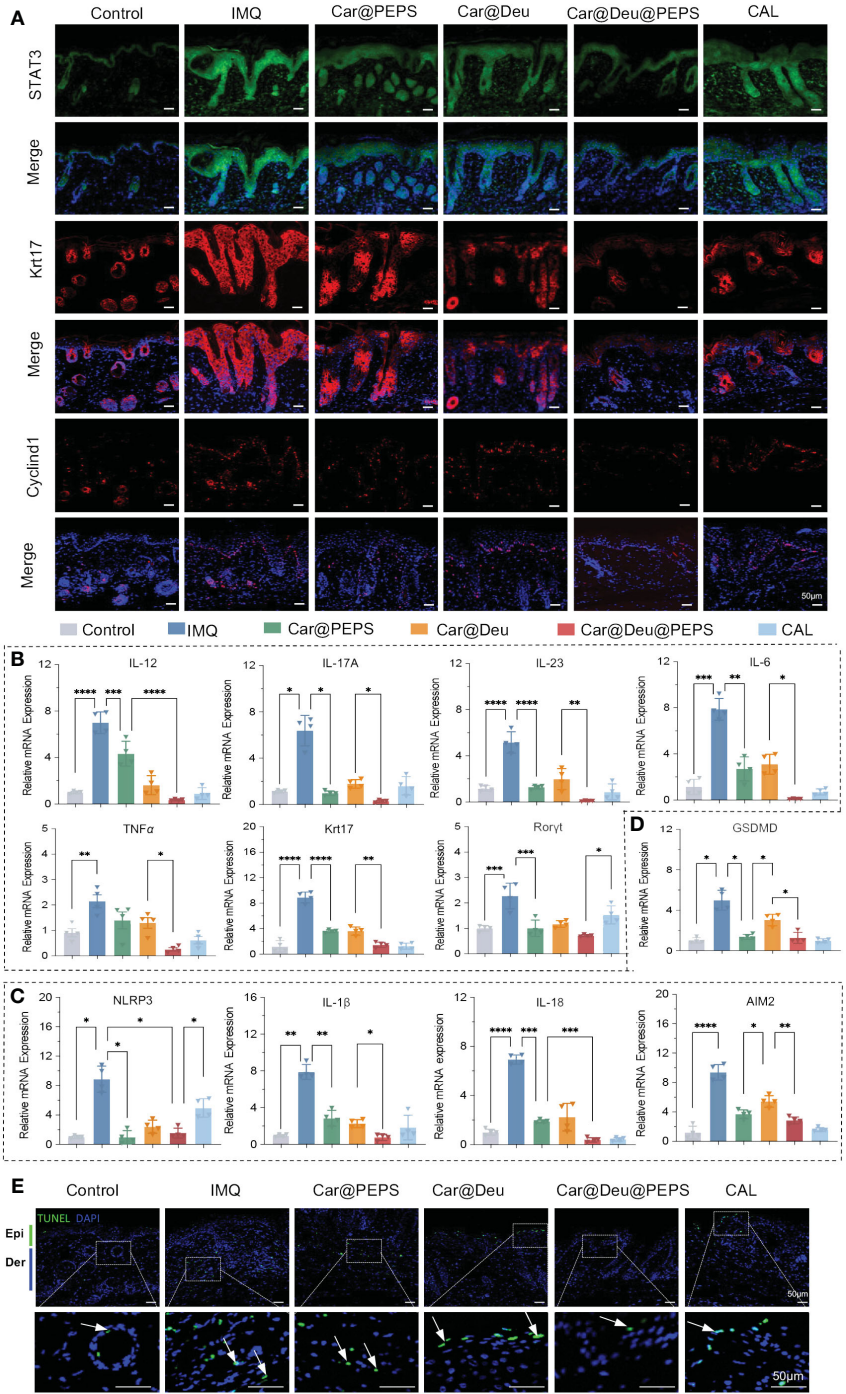
Deu@PEPS suppresses keratin 17 and Cyclin D1 by STAT3 pathway and improves the transcriptional phenotype of psoriasiform *in vivo*. **(A)** Immunofluorescence analysis in skin tissue sections to determine the expression of STAT3,K17 and Cyclin D1 (n = 3). **(B)** QRT-PCR of relative mRNA expression of IL-23-IL-17A axis (*IL-12, IL-17A, IL-23*), innate immunity (*IL-6* and *TNFα*), proliferation gene (*Krt17*) and Th17 differentiation gene (*Rorγt*). **(C)** QRT-PCR of relative mRNA expression of inflammasome related genes (*AIM2, NLRP3, IL-1β, IL-18*). **(D)** QRT-PCR of relative mRNA expression of pyroptosis related gene (*GSDMD*). **(E)** Representative images of TUNEL positive cells (apoptotic cells) in the skin lesions with different treatments (scale bar: 50 μm, n = 3). Epi, epidermis; Der, dermis; IMQ, imiquimod. *∗p < 0.05, ∗∗p < 0.01, ∗∗∗p < 0.001, ∗∗∗∗p < 0.0001*.

Autoimmune feedback loop mediated by K17 is vital in psoriasis both as the inflammation responder and adjuster (44). Accordingly, we investigated K17 and Cyclin D1 under increased STAT3 expression in psoriatic skin samples by immunofluorescence. IMQ remarkably promoted the expression of K17 and Cyclin D1 by inducing STAT3 expression, whereas the topical administration of Car@Deu@PEPS remarkably reversed these trends. Car@Deu alone also reduced the expression of these factors to some extent, which was likely owing to inhibition of STAT3 as a TYK2 inhibitor (**Figure 7A**, **Additional file 1: Figure S9**). The results of qRT-PCR analysis showed that the Deu@PEPS topical formula significantly reduced the mRNA expression of psoriatic elements, including cytokines in the IL-23/IL-17 axis (*IL-12, IL-17A, IL-23*), cytokines associated with innate immunity (*IL-6* and *TNF*α), edigree specific transcription factors of Th17 cell differentiation (*RORγt*), and the keratinocyte proliferation marker (*Krt17*) compared to those of the other groups (**Figure 7B**), including the positive control group.

IMQ treatment upregulated the mRNA levels of *NLRP3*, *IL-1β* and *IL-18*, indicating that the inflammasome of NLRP3 was activated in the skin of IMQ-induced psoriatic mice. *AIM2*, which was associated with the assembly of NLRP3 and the secretion of the pro-inflammatory cytokines IL-1β and IL-18, was also upregulated in the IMQ group. PEPS could cooperate with deucravacitinib to reduce the IMQ-induced elevations in the mRNA expression of inflammasomes and related cytokines by its antioxidative effects in skin lesions (**Figure 7C**). Deu@PEPS also had an effect on pyroptosis by reducing the *GSDMD* expression level that was elevated in the IMQ group (**Figure 7D**). Finally, terminal deoxynucleotidyl transferase dUTP nick-end labeling (TUNEL) staining was conducted to detect pyroptotic cascades that lead to DNA fragmentation. Consistent with the results of qRT-PCR, the results of the TUNEL assay revealed that the number of apoptotic cells in the IMQ mice was higher than that of the control group; however, no increase in TUNEL-positive cells was detected after treatment with Deu@PEPS (**Figure 7E**, **Additional file 1: Figure S10**).

### Skin irritation and biocompatibility of SPMs hydrogels *in vivo*

**Figure 8:**
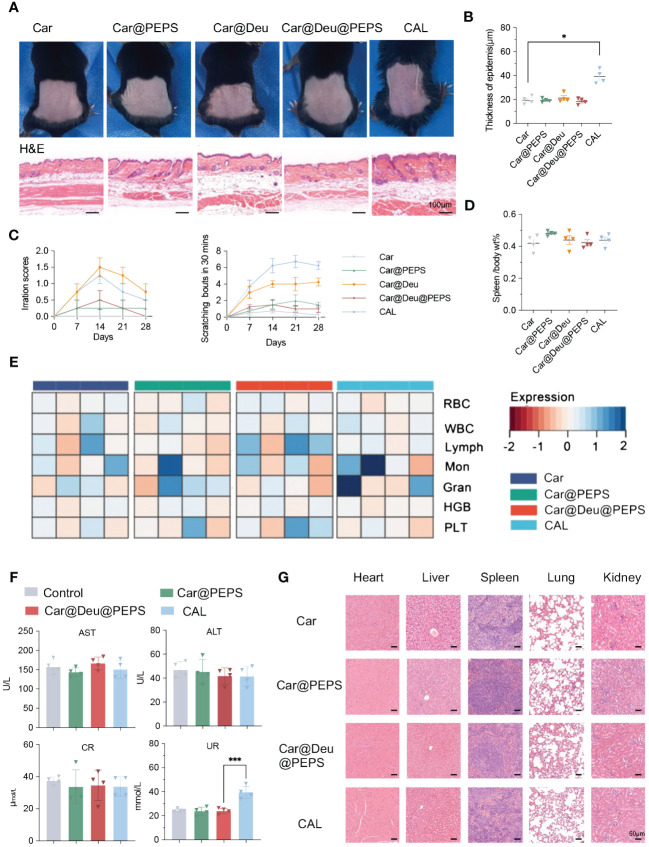
Evaluation of skin irritation and toxicological studies treatment with various hydrogels for transdermal therapy on healthy mice. **(A)** Photographs of skin appearances and H&E staining of dorsal skins (n = 3, scale bar:100 μm). **(B)** Thickness of epidermis on H&E staining of different groups analysed by Image J (n = 3). **(C)** The sensitization of skin by irritation score and scratching behaviour every 7days. **(D)** The spleen/body wt% of different groups. **(E)** Analysis of red blood cells (RBC), white blood cells (WBC), lymphocytes (Lymph), Monocytes (Mon), granulocyte (Gran), hemoglobin (HGB) and platelets (PLT) with different treatments. **(F)** Evaluation of the liver and kidney functions by serum biochemical indicators [aspartate transaminase (AST), alanine transaminase (ALT), creatinine (CR) and UREA (UR)] (n = 4). **(G)** Representative H&E-stained sections of the main organs (scale bars: 50 μm). *∗p < 0.05, ∗∗∗p < 0.001*.

For transdermal delivery of nanomedicines, skin irritation and nanomedicine toxicity assessment are essential for their clinical translation (45). Previous work has demonstrated that PEPS exerts a non-inflammatory (46). To confirm the feasible clinical application of the SPMs hydrogel formulation, we assessed their potential for irritation according to the skin appearance and histological examination after application to the back skin of normal mice continuing 28 days. The surface of mice showed mild erythema in the Car@Deu group and certain level of skin atrophy in CAL group, whereas atrophy was negligible in the other treatment groups(**Figure 8A**). Skin H&E staining showed no apparent pathological changes, except for epidermal thickening detected in the CAL group, compared with the Car group (**Figure 8B**). CAL and Car@Deu treatments led to increased scratching bouts compared to the mice in the other groups (**Figure 8C**). There were no evident changes in appearance of the spleen or in the spleen/body wt% of the different groups, indicating no significant activation of the immune system for long-term toxicity (**Figure 8D**, **Additional file 1: Figure S11**). Over the 28 days of treatment, the CAL and Car@PEPS groups had the least and greatest weight change compared to the initial weight, respectively (**Additional file 1: Figure S12**).

Additionally, biological safety evaluation was preformed by blood routine examination and serum biochemical indicators. According to **Figure 8E**, compared to Car only, both Car@PEPS and Car@Deu@PEPS didnot affect blood cell count, including red and white blood cells, monocytes (Mon), lymphocytes (Lymph), granulocytes (GRAN), platelets (PLT) and hemoglobin (HGB). Simultaneously, both Car@PEPS and Car@Deu@PEPS did not exhibit any hepatotoxicity or nephrotoxicity (**Figure 8F**) except for long term CAL. Histological examination of the main organs showed no obvious pathological changes such as necrosis, hyperplasia, or inflammatory cell infiltration (**Figure 8G**). Therefore, these results could reasonably indicate that Car@Deu@PEPS is a biocompatible product with minimal skin irritation and negligible toxicity, demonstrating its further potential for achieving the topical application of deucravacitinib in clinical practice.

## Discussion

Clinically, topical medications are needed for most mild to moderate psoriasis patients, however there is still a lack of effective topical medications. JAK inhibitors have become a powerful weapon for dermatologists in the treatment of various dermatological diseases in recent years, especially in critically ill patients. However, the side effects of JAK inhibitors, especially thrombosis, anemia, bone marrow transplantation and other effects on the blood system, most of which are dose-dependent, have limited their utilization (47, 48). Considering the clinical population applicability and the high oxidative stress microenvironment of psoriatic lesions, our study aims to explore new delivery patterns and transdermal targeted synergistic effects of JAK inhibitors, to reduce the dosage and adverse effects of oral JAKi while achieving synergistic therapeutic effects through transdermal administration in the overloaded ROS microenvironment of psoriasis.

Previous studies revealed that the production of excess ROS in psoriatic lesions disequilibrates the redox system of keratinocytes to activate pro-inflammatory signaling cascades, leading to the hyperproliferation of keratinocytes (9). Long-term oxidative stress induces a transformation of the mitochondrial membrane redox potential, leading to mtDNA damage (49).We successfully established the psoriasiform and oxidative stress HaCaT model induced by TNF-α and IL-17A and the results certified that Deu@PEPS abrogates the psoriatic hyperproliferation of keratinocytes by restoring ROS-mediated mitochondrial disfunction compared to deucravacitinib alone. In other words, deucravacitinib has a more effective anti-proliferation role under the synergy of PEPS supramolecular micelles, indicating that the superior inhibitory effect of Deu@PEPS could be attributed to the enhanced uptake and prolonged release profile of the drug.

*In vivo*, the skin permeability efficacy used kallikrein-8 (KLK8) as a marker to distinguish between the epidermis and dermis (50, 51). Our findings demonstrates that Car@Deu@PEPS-Rhb has a longer residence time in skin lesions of the psoriatic mice model, which may be related to the hyperproliferative properties of the stratum corneum and the overproduction of ROS. In addition, the fluorescence observed in the PBS group may be attributed to the effect of the excitation and emission wavelength range of Rhb on the blood, but this difference can be calculated. This finding is consistent with previous studies showing that 100–200 nm SPMs could penetrate the skin layer with prolonged residence time (52, 53). Therefore, Deu@PEPS had longer-lasting dermal retention in psoriatic model, demonstrating the therapeutic targeting capacity.

In IMQ-induced psoriatic dermatitis, we certified Car@Deu@PEPS exerts a remarkable anti-psoriatic therapeutic effect to improve psoriatic clinical and histological features and it can minimize the dose of deucravacitinib required in transdermal delivery. We utilized carpotriol as the positive control rather than oral deucravacitinib for obvious diversity in pharmacokinetics. We deem that transdermal delivery has the advantages in targeting ROS microenvironment of psoriatic lesions. A recent study reported that oral large dose deucravacitinib (30 mg/kg QD) showed the similar equivalent efficacy to ustekinumab rather than medium dose deucravacitinib (10 mg/kg QD) in IL-23-driven psoriasis-like model (15). IMQ affects mitochondrial ROS, which are required for the activation of immune cells contributing to the severe inflammation in the development of psoriatic dermatitis (54). Deu@PEPS performed remarkable antioxidative effects by increasing the content of antioxidative enzymes and restraining the expression of lipid peroxidative product. The antioxidative effects of PEPS might be attributed to activation of the HO-1/NRF2-mediated antioxidant pathway and promoted the translocation of SOD2 to clear ROS (40, 55).

K17 activates the transcription factor STAT3 under induction of deleterious ROS production in keratinocytes along with Cyclin D1, leading to the hyperproliferation of keratinocytes (56–58). A previous study suggested that STAT3 activation enables its nuclear localization, which triggers the transcription of downstream genes such as *TNF-α, IL-6, IL-17* and *IL-22* (59). STAT3 could exert functions by regulating mitochondrial dysfunction. ROS produced by mitochondria could been influenced by mitochondrial STAT3, which regulates electron transport chain (ETC) activity and cellular respiration in particular with the synthesis of ATP (60, 61). ROS and mitochondrial dysfunction can trigger activation of NLRP3 inflammasomes (62). NLRP3 activation leads active assembles of IL-18 and IL-1β, regulates the splicing of gasdermin D (GSDMD) into fragments to induce pyroptosis (63, 64). Our findings demonstrated that compared to treatment with deucravacitinib alone, Deu@PEPS is more effective in ameliorating the induction of multiple cytokines and important molecular features associated with psoriasis by STAT3-Krt17-Cyclin D1 pathway. And overloaded ROS in IMQ skin lesions activate the NLRP3 inflammasome to subsequently trigger pyroptosis, which could be reversed by Deu@PEPS administration. Overall, Deu@PEPS transdermal delivery showed an optimized therapeutic effect at both the phenotypic and molecular levels with good biocompatibility with long-term use.

Our study also has some limitations. Firstly, PEPS is not the original ROS-related nano-delivery system. But given its low toxicity and lack of inflammatory response, we believe it is still necessary to explore its clinical application for targeting the overloaded ROS environment in psoriasis. Secondly, though IMQ-induced psoriasis-like model is widely used, there is still a big gap between its acute inflammation and the chronic inflammation of psoriasis patients, which is still needed to further exploring. Finally, we believe that topical administration can reduce the dose of orally administered drugs and JAK-related side effects, but further validation is needed to determine whether it can indeed reduce JAK-related side effects.

In conclusion, we focused on advanced TYK2 inhibitor to develop a new ROS-responsive topical formula of deucravacitinib based on PEPS micelles with inherent antioxidative capacity for psoriasis treatment. Therefore, we improved the formula through nanotechnology to reduce the dosage to expand the applicable population and promote clinical application. We reckon that the new topical formula of Deu@PEPS offers a more patient-acceptable therapy method with fewer adverse effects owing to the low therapeutic dose of deucravacitinib. The use of such an ROS-responsive nanoplatform for drug formulation modification may provide a new strategy for not only the delivery of deucravacitinib but also other small-molecule drugs in the JAK family, which can offer a promising clinical translation strategy.

## Data availability statement

The original contributions presented in the study are included in the article/**Supplementary Material**. Further inquiries can be directed to the corresponding author.

## Ethics statement

The animal study was approved by Animal Ethics Committee of Xi’an Jiaotong University. The study was conducted in accordance with the local legislation and institutional requirements.

## Author contributions

ZL: Data curation, Methodology, Supervision, Writing – original draft, Writing – review & editing. LY: Formal analysis, Investigation, Methodology, Project administration, Writing – original draft, Writing – review & editing. FT: Investigation, Project administration, Software, Supervision, Writing – review & editing. QM: Data curation, Methodology, Resources, Visualization, Writing – review & editing. LG: Methodology, Project administration, Writing – review & editing. ZM: Formal analysis, Project administration, Writing – original draft. TH: Methodology, Software, Writing – review & editing. QL: Data curation, Methodology, Writing – review & editing.
