# Supplementary material for: Reactive oxygen species-responsive supramolecular deucravacitinib self-assembly polymer micelles alleviate psoriatic skin inflammation by reducing mitochondrial oxidative stress
Source: Front Immunol. 2024 May 10;15:1407782. doi: 10.3389/fimmu.2024.1407782 (PMC11116664; doi:10.3389/fimmu.2024.1407782)

Reactive oxygen species-responsive supramolecular deucravacitinib self-assembly polymer micelles alleviate psoriatic skin inflammation by reestablishing mitochondrial function

Leiqing Yao[1], Faming Tian[2], Qinqin Meng[1], Lu Guo[1], Zhimiao Ma[1], Ting Hu[1], Qiongwen Liang[1], Zhengxiao Li[1] *

[1]Department of Dermatology, The Second Affiliated Hospital of Xi'an Jiaotong University, Xi'an 710004, China.

[2]Medical Research Center, North China University of Science and Technology, Tangshan Hebei 063000, China.

* Correspondence: lizhengxiao1979@163.com (Z. Li)

Supplymentary figures

Figure S1. 1H nuclear magnetic resonance (1H NMR) spectra of the poly (ethylene glycol)-block-poly propylene sulfide (PEPS) recorded by spectroscopy.

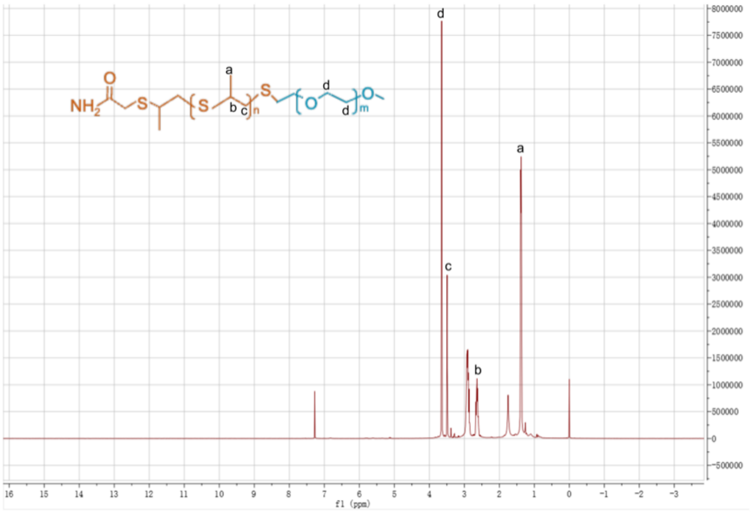

Figure S2. Characterization of Rhodamine b-labeled PEPS (Rhb-PEPS) by UV spectroscopy and fluorescence spectroscopy (excitation wavelength of 500-530nm and emission wavelength of 550-650 nm) analyses.

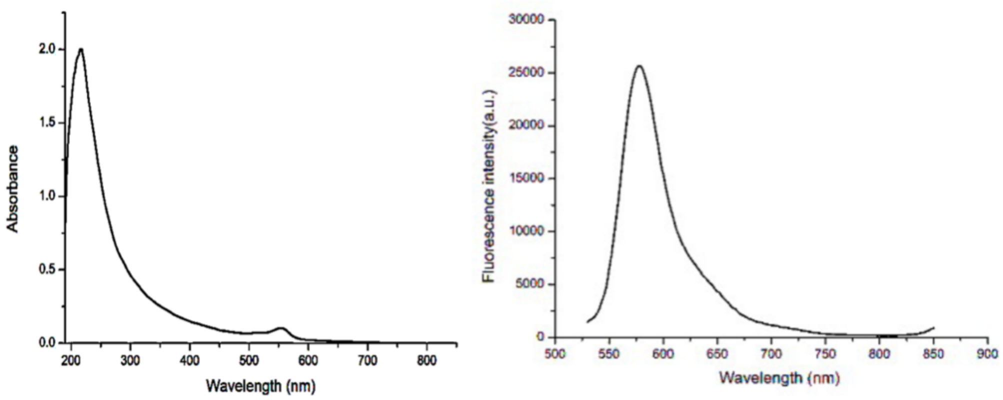

Figure S3. The micelle stability of Deu@PEPS within 1 months at room temperature detected by Dynamic light scattering (DLS) (n = 3).

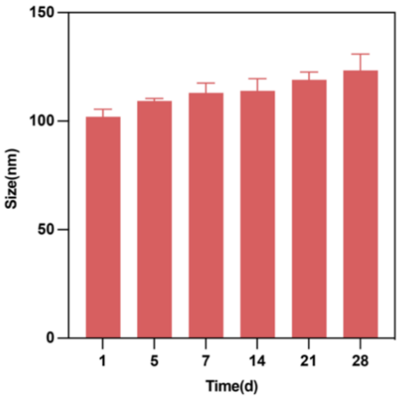

Figure S4. Fluorescent images of cellular ROS level (DCFH-DA, green) in Deu@PEPS or deucravacitinib pretreated HaCaT keratinocytes incubated with IL-17A and TNFα or H_2O_2 (200 μM) and quantitative analysis of relative fluorescent areas in four groups (scale bar: 100 μm, n = 3).

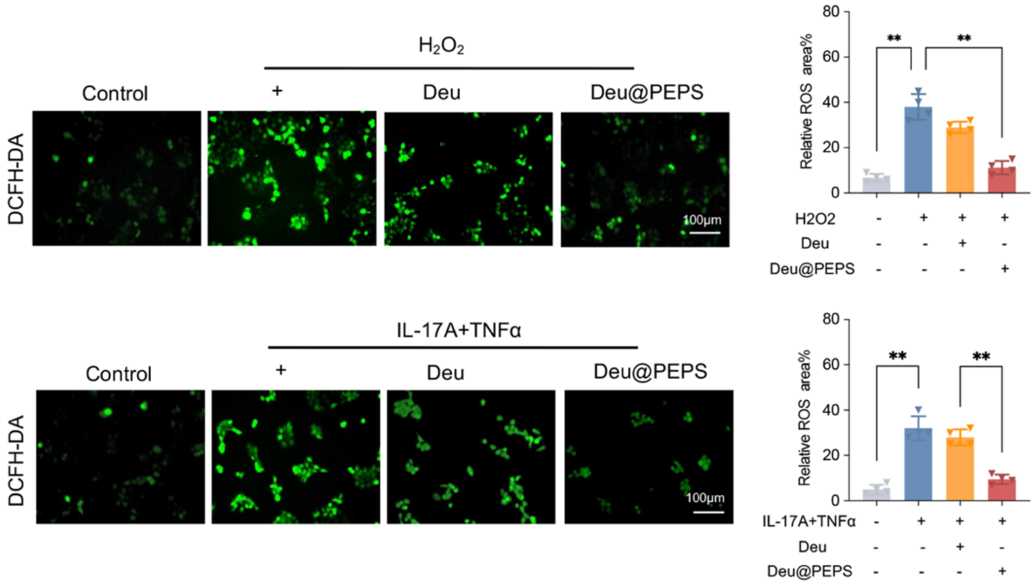

Figure S5. Total antioxidant capacity (T-AOC) and superoxide dismutase (SOD) level in HaCaT after different treatments (n = 4). $*p < 0.05$, $**p < 0.01$, $***p < 0.001$.

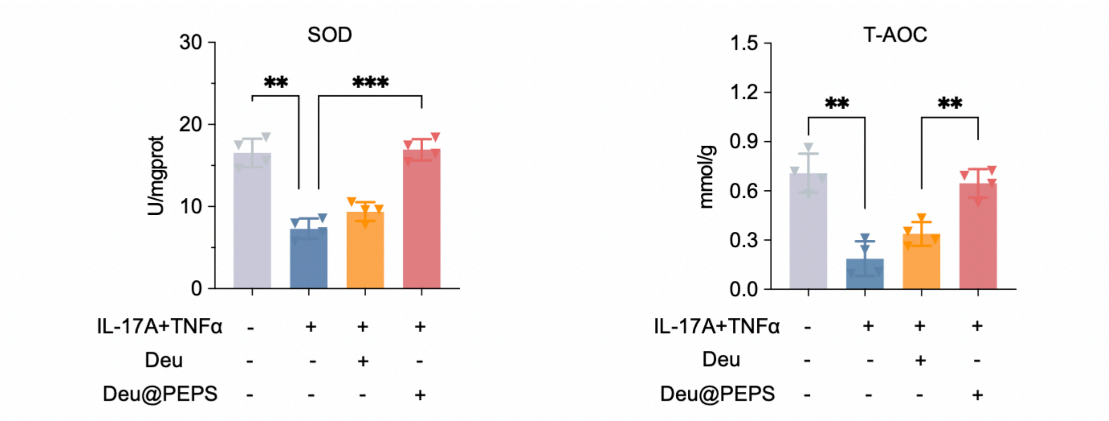

Figure S6. A pilot study of topical therapeutic effects of Deu@PEPS in IMQ-induced psoriatic dermatitis. **A** Representative clinical pictures of mice treated with various formulas on day 7 before harvesting back skin. Mice were depilated on day 0 and divided into six groups (n = 3) including control, IMQ and PEPS (10 mg/kg, QD), Deu(5 mg/kg, QD), Deu@PEPS (10 mg/kg PEPS and 0.45mg/kg Deu, QD) and Deu@PEPS (10 mg/kg PEPS and 0.45mg/kg Deu, BID). **B** Spleen appearances of different groups. **C** Hematoxylin and eosin staining (H&E) of day 7-harvest mouse skin(scalr bars:100 μm,n = 3). **D** Total psoriasis Area and Severity Index (PASI) scores of different groups in the dorsal skin for 7 days. **E** The radio of spleen weight to body (spleen body wt%) of different groups (n = 3). IMQ: imiquimod; **p < 0.01.

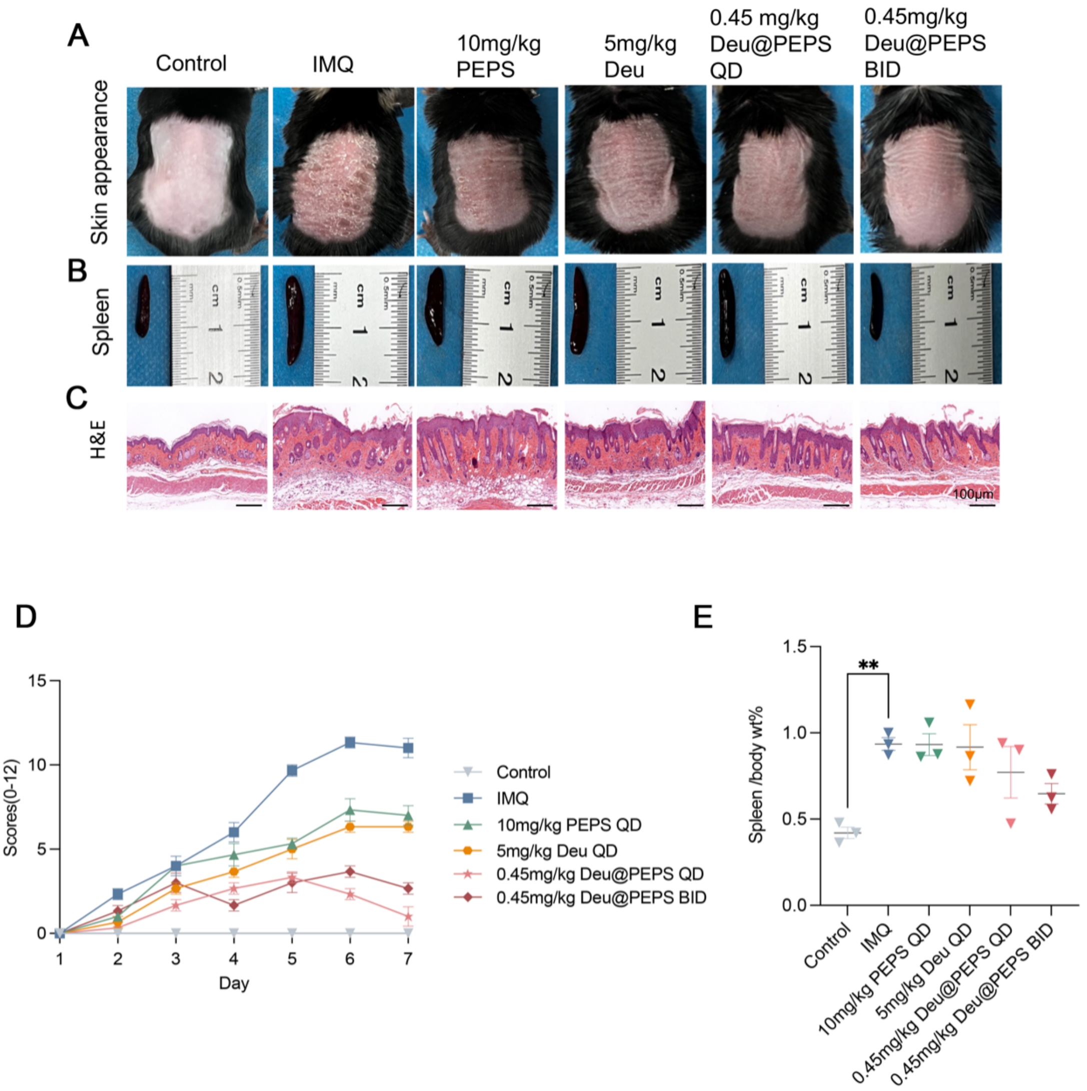

Figure S7. Weight changes on day7 of different groups (n = 6). **p < 0.01, ***p < 0.001, **** p < 0.0001.

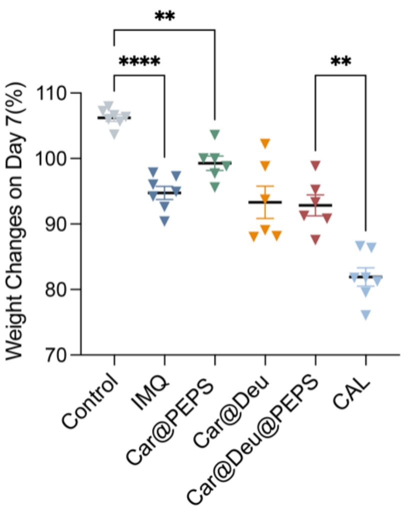

Figure S8. Toxicological studies of different formula hydrogels topical therapy on IMQ-induced psoriatic dermatis (duration: six days). **A** Representative Hematoxylin and eosin (H&E) staining sections of the main organsof each group (scale bar: 100 μm, n = 3). **B** Evaluation of hepatotoxicity and nephrotoxicity by serum biochemical tests of aspartate transaminase (AST), alanine transaminase (ALT), creatinine (CR), UREA (UR) (n = 4).

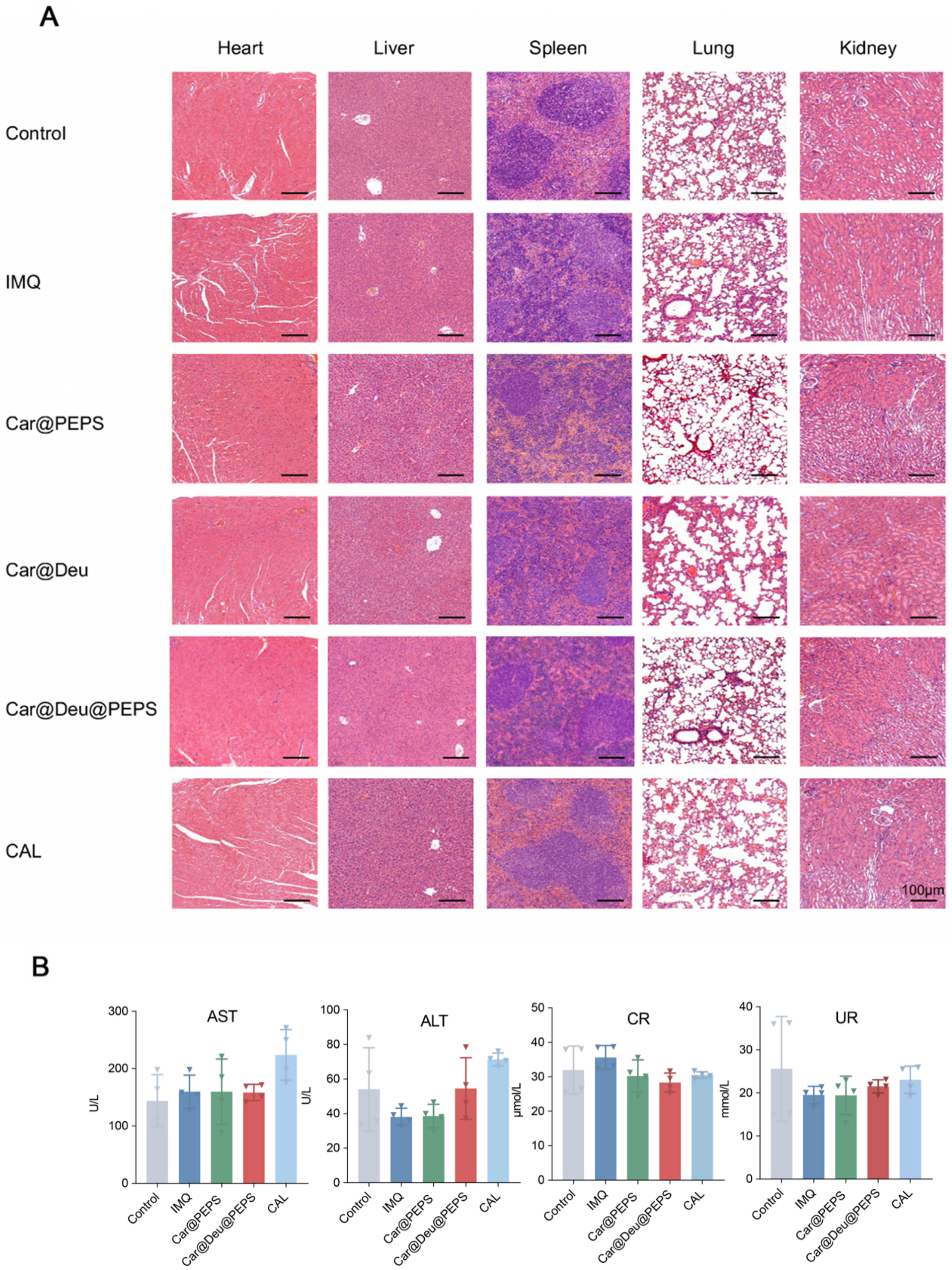

Figure S9. Immunofluorescence (IF) of DAPI in skin tissue sections of STAT3, Krt17 and Cyclin D1 (scale bar: 50 μm, n = 3)

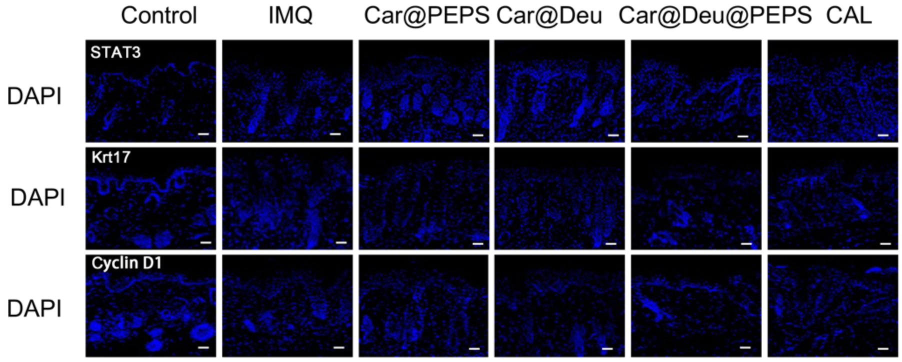

Figure S10. Representative images of DAPI in TUNEL positive cells (apoptotic cells) in the skin lesions with different treatments. (scale bar: 50 μm, n = 3).

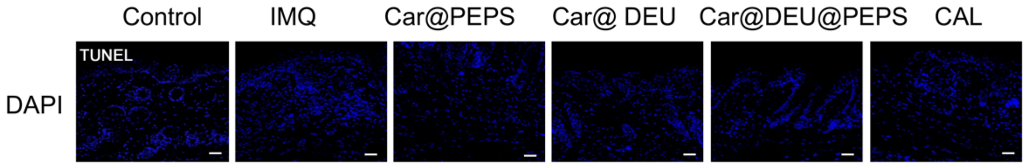

Figure S11. Spleen photographs of different groups continuing 28 days (n = 4).

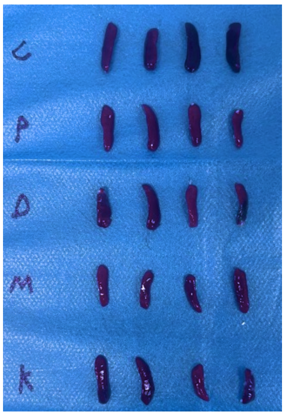

Figure S12. Weight changes of different groups during 0- 28days (n = 4).

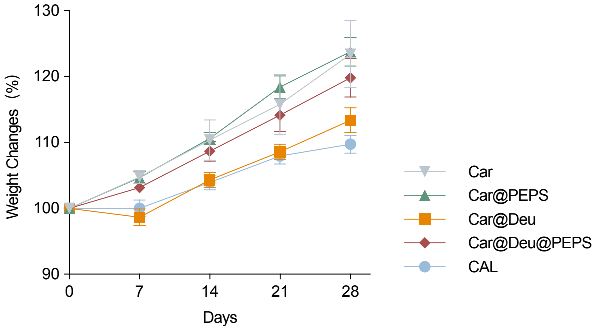

Supplement: Supplementary file 1 [file DataSheet_1.pdf]
