# Supplementary material for: Reactive oxygen species-responsive supramolecular deucravacitinib self-assembly polymer micelles alleviate psoriatic skin inflammation by reducing mitochondrial oxidative stress
Source: Front Immunol. 2024 May 10;15:1407782. doi: 10.3389/fimmu.2024.1407782 (PMC11116664; doi:10.3389/fimmu.2024.1407782)
Supplement: Supplementary file 2 [file Table_1.pdf]

Reactive oxygen species-responsive supramolecular deucravacitinib self-assembly polymer micelles alleviate psoriatic skin inflammation by reestablishing mitochondrial function

Leiqing Yao[1], Faming Tian[2], Qinqin Meng[1], Lu Guo[1], Zhimiao Ma[1], Ting Hu[1], Qiongwen Liang[1], Zhengxiao Li[1] *

[1]Department of Dermatology, The Second Affiliated Hospital of Xi'an Jiaotong University, Xi'an 710004, China.

[2]Medical Research Center, North China University of Science and Technology, Tangshan Hebei 063000, China.

* Correspondence: lizhengxiao1979@163.com (Z. Li)

Supplymentary Tables

Table S1. The particle sizes and zata potentials of PEPS-Rhb (n = 3).

	**Hydrodynamic size (nm)**	**PDI**	**Zeta potential (mV)**
**1**	105	0.17	-17.63
**2**	107	0.16	-17.08
**3**	108	0.17	-14.04
**Average**	106.67±1.33	0.167±0.003	-16.25±2.21

Table S2. Primers used in mtDNA copy number assay.

**Gene name**	**Primer sequence (5'-3')**	**Species**
hB2M-F	TGTTCCTGCTGGGTAGCTCT	Human
hB2M-R	CCTCCATGATGCTGCTTACA	Human
mtND1 -F	CACTTTCCACACAGACATCA	Human
mtND1-R	TGGTTAGGCTGGTGTTAGGG	Human

Table S3. The sequence of primers used in qRT-PCR.

Gene name	Primer sequence (5'-3')	Species
***IL-12*-F**	TTGAACTGGCGTTGGAAGCACG	Mouse
***IL-12*-R**	CCACCTGTGAGTTCTTCAAAGGC	Mouse
IL-17A-F	TTTAACTCCCTTGGCGCAAAA	Mouse
***IL-17A*-R**	CTTTCCCTCCGCATTGACAC	Mouse
IL-23-F	GCACCTGCTTGACTCTGACATCTTC	Mouse
***IL-23*-R**	TGGCTGGAGGAGTTGGCTGAG	Mouse
***IL-1*β-F**	CTCGCAGCAGCACATCAACAAG	Mouse
***IL-1*β-R**	CCACGGGAAAGACACAGGTAGC	Mouse
***IL-18*-F**	AAAGTGCCAGTGAACCCCAGAC	Mouse
***IL-18*-R**	AGAGAGGGTCACAGCCAGTCC	Mouse
IL-6-F	TGGGACTGATGCTGGTGACAAC	Mouse
***IL-6*-R**	AAGCCTCCGACTTGTGAAGTGG	Mouse
TNF*α*-F	CCCTCACACTCAGATCATCTT	Mouse
***TNF*α-R**	GCTACGACGTGGGCTACAG	Mouse
***Krt17*-F**	ACCATCCGCCAGTTTACCTC	Mouse
Krt17-R	CTACCCAGGCCACTAGCTGA	Mouse
Rorgt-F	GACCCACACCTCACAAATTGA	Mouse
Rorgt-R	AGTAGGCCACATTACACTGCT	Mouse
AIM2-F	CTGGACCACATCACGGAGGAAG	Mouse
AIM2-R	GCCGCACCTGCACTTTGAATC	Mouse
NLRP3-F	GATGGGTTTGCTGGGATA	Mouse
NLRP3-R	AGCTGCGTGTAGCGACTG	Mouse
Gsdmd-F	CCA TCG GCC TTT GAG AAA GTG	Mouse
Gsdmd-R	ACA CAT GAA TAA CGG GGT TTC C	Mouse
HO-1-F	AAGACCGCCTTCCTGCTCAAC	Mouse
HO-1-R	TCTGACGAAGTGACGCCATCTG	Mouse

NRF2-F	AGCACAGCCAGCACATTCTCC	Mouse
NRF2-R	GACCAGGACTCACGGGAACTTC	Mouse
SOD1-F	CAAGGCTGTACCAGTGCAGGAC	Mouse
SOD1-R	CAGTCACATTGCCCAGGTCTCC	Mouse
SOD2-F	AGCCTCCCAGACCTGCCTTAC	Mouse
SOD2-R	CTTCTCCTCGGTGGCGTTGAG	Mouse
IL-12-F	AGATGTACCAGGTGGAGTTCAAGAC	Human
IL-12-R	GGCCTGCATCAGCTCATCAATAAC	Human
IL-17A-F	CATAGCAGGCACAAACTCATCCATC	Human
IL-17A-R	CAGCAGTAGCAGTGACACCAATG	Human
IL-23-R	CCACTGGGAGACTCAGCAGATTC	Human
IL-23-F	CGAAGGATTTTGAAGCGGAGAAGG	Human
TNFα-F	CCGAGTGACAAGCCTGTAGCC	Human
TNFα-R	TGAAGAGGACCTGGGAGTAGATGAG	Human
***GAPDH*-F**	CGAAGGTGGAGTCAACGGATTT	Human
***GAPDH*-R**	ATGGGTGGAATCATATTGGAAC	Human
***GAPDH*-F**	AGGTCGGTGTGAACGGATTTG	Mouse
***GAPDH*-R**	TGTAGACCATGTAGTTGAGGTCA	Mouse
b-ACTIN-F	TGCTGTCCCTGTATGCCTCTG	Mouse
b-ACTIN-R	TGATGTCACGCACGATTTCC	Mouse
